# Supplementary material for: Prognosis and Survival in Idiopathic Pulmonary Fibrosis in the Era of Antifibrotic Therapy in Italy: Evidence from a Longitudinal Population Study Based on Healthcare Utilization Databases
Source: Int J Environ Res Public Health. 2022 Dec 12;19(24):16689. doi: 10.3390/ijerph192416689 (PMC9779053; doi:10.3390/ijerph192416689)
Supplement: Supplementary file 1 [file ijerph-19-16689-s001.zip › ijerph-2008045-supplementary.pdf]

Table S1. List of ICD-9-CM and ATC codes used to detect pulmonary malignant neoplasm.

Data source	ICD-9-CM/ATC codes
Hospital Discharge Records	ICD-9-CM: 162, 162.2, 162.3, 162.4, 162.5, 162.8, 162.9, V58.0, in primary or secondary diagnoses;<br>ICD-9-CM: 32.23, 32.25, 32.26, 32.28, 32.29, 32.3, 32.4, 32.5, 92.2x, in primary or secondary procedures
Drug prescriptions database	ATC: L01XE03, L01XE02, L01XE13, L01XE47, L01XE35, L01XE16, L01XE36, L01XE28, L01XE44, L01XE43
Outpatient care database	ICD-9-CM: 99.25

Table S2. List of ICD-9-CM codes used to identify acute exacerbation of IPF.

ICD-9-CM code	Code description
465	Acute upper respiratory infections of multiple or unspecified sites
466	Acute bronchitis and bronchiolitis
478.9	Other and unspecified diseases of upper respiratory tract
480	Viral pneumonia
481	Pneumococcal pneumonia [Streptococcus pneumoniae pneumonia]
482	Other bacterial pneumonia
483	Pneumonia due to other specified organism
484	Pneumonia in infectious diseases classified elsewhere
485	Bronchopneumonia, organism unspecified
486	Pneumonia, organism unspecified
487	Influenza
490	Bronchitis, not specified as acute or chronic
494	Bronchiectasis
510	Empyema
511	Without mention of effusion or current tuberculosis
511.1	With effusion, with mention of a bacterial cause other than tuberculosis
511.8	Other specified forms of effusion, except tuberculous
513	Abscess of lung and mediastinum
517	Lung involvement in conditions classified elsewhere
518.81	Acute respiratory failure
518.82	Other pulmonary insufficiency, not elsewhere classified
518.84	Acute and chronic respiratory failure, Acute on chronic respiratory failure
518.89	Other diseases of lung, not elsewhere classified
519.1	Other diseases of trachea and bronchus, not elsewhere classified
519.11	Acute bronchospasm
786	Respiratory abnormality, unspecified
786.01	Hyperventilation
786.05	Shortness of breath
786.07	Wheezing
786.09	Other Respiratory: distress, insufficiency
786.2	Cough
786.3	Hemoptysis
786.4	Abnormal sputum
786.5	Chest pain
786.7	Abnormal chest sounds
786.9	Other symptoms involving respiratory system and chest
